# Integrated single-cell RNA-seq and DNA methylation reveal the effects of air pollution in patients with recurrent spontaneous abortion

**DOI:** 10.1186/s13148-022-01327-2

**Published:** 2022-08-23

**Authors:** Weiqiang Zhu, Yan Gu, Min Li, Zhaofeng Zhang, Junwei Liu, Yanyan Mao, Qianxi Zhu, Lin Zhao, Yupei Shen, Fujia Chen, Lingjin Xia, Lin He, Jing Du

**Affiliations:** 1 NHC Key Lab of Reproduction Regulation (Shanghai Institute for Biomedical and Pharmaceutical Technologies), School of Pharmacy, Fudan University, 2140 Xietu Road, Shanghai, 200032 China; 2 Department of Gynecology and Obstetrics Outpatient, Second Hospital of Tianjin Medical University, Tianjin, China; 3 Institutes of Biomedical Sciences, The State Key Laboratory of Genetic Engineering, Fudan University, Shanghai, 200032 China; 4 Bio-X Center, Key Laboratory for the Genetics of Developmental and Neuropsychiatric Disorders, Ministry of Education, Shanghai Jiao Tong University, Shanghai, 200030 China

**Keywords:** RSA, scRNA-seq, RRBS, Air pollutants, PLS-PM

## Abstract

**Background:**

Maternal air pollutants exposure is associated with a number of adverse pregnancy outcomes, including recurrent spontaneous abortion (RSA). However, the underlying mechanisms are still unknown. The present study aimed to understand the mechanism of RSA and its relationship with air pollution exposure. We compared data of decidual tissue from individuals with induced abortions and those with RSA by bulk RNA sequencing (RNA-seq), reduced representation bisulfite sequencing (RRBS), and single-cell RNA sequencing (scRNA-seq). Differentially expressed genes (DEGs) were verified using RT-qPCR and pyrosequencing. A logistic regression model was used to investigate the association between air pollutants exposure and RSA.

**Results:**

We identified 98 DEGs with aberrant methylation by overlapping the RRBS and RNA-seq data. Nineteen immune cell subsets were identified. Compared with normal controls, NK cells and macrophages accounted for different proportions in the decidua of patients with RSA. We observed that the methylation and expression of *IGF2BP1* were different between patients with RSA and controls. Furthermore, we observed significant positive associations between maternal air pollutants exposure during the year prior to pregnancy and in early pregnancy and the risk of RSA. Mediation analyses suggested that 24.5% of the effects of air pollution on the risk of RSA were mediated through *IGF2BP1* methylation.

**Conclusion:**

These findings reveal a comprehensive cellular and molecular mechanism of RSA and suggest that air pollution might cause pregnancy loss by affecting the methylation level of the *IGF2BP1* promoter.

**Supplementary Information:**

The online version contains supplementary material available at 10.1186/s13148-022-01327-2.

## Background

Ambient air pollution has become a global environmental threat [1]. The primary air pollutants mainly include particulate matter (PM), nitrogen dioxide (NO_2_), carbon monoxide (CO), sulfur dioxide (SO_2_), and ozone (O_3_), among which PM remains one of the most harmful forms, contributing to more than 4.2 million premature mortalities [2]. Evidence from animal and human studies suggests that exposure to air pollution can reduce fertility rates and increase the risk of miscarriage [3]. When the concentration of ambient PM is more than 40 µg/m^3^, an estimated 3.5 million pregnant women experience a miscarriage per year in South Asia [4]. Besides, exposure to NO_2_ during early pregnancy was associated with increased odds of spontaneous abortion in linear dose–response manners [5]. A case–crossover analysis reported that PM10 (particles < 10 μm), PM2.5–10 (particles between 2.5 μm and 10 μm), and PM2.5 exposure were positively associated with an increased risk of spontaneous abortion in the USA [6]. CO exposure during the first trimester of pregnancy was linked to an increased risk of spontaneous abortion in case–control research from Iran, which reported a 95 percent increase in spontaneous abortions in cases compared to controls [7]. In some cities of China such as Jiangsu, Beijing, and Wuhan, the researchers also found that maternal exposure to air pollution was significantly associated with an increased risk of incident spontaneous pregnancy loss [8–10]. Although ambient air pollution has been linked with spontaneous abortion [11, 12], the relationship between recurrent spontaneous abortion (RSA) and air pollution and its underlying molecular mechanism is not well established.

RSA, one of the most common complications of pregnancy, is usually defined as three or more consecutive spontaneous abortions with the same spouse [13]. Its incidence is 2–4% among clinically recognized pregnancies [14]. RSA is a multifactorial disease condition, which has been associated with environmental pollution, chromosomal abnormalities, genital tract anatomic abnormalities, endocrine disorders, autoimmunological factors, and infectious diseases. To date, several foundational studies have employed single-cell RNA sequencing (scRNA-seq) technology to investigate the cellular composition and inter-cellular communication events at the maternal–fetal interface in patients with RSA [15–17]. Advancements in scRNA-seq have greatly facilitated the development of novel approaches to improve targeted therapies and precision medicines [18]. However, these studies only analyzed the pathological mechanism of RSA from the perspective of immunology and did not completely construct all cell atlas of RSA patients. A more comprehensive cell atlas is therefore needed and provides new insights and bases for studying the pathogenesis and the clinical treatment of RSA.

Accumulating evidence indicates that exposure to air pollution, especially PM2.5, causes changes in DNA methylation both in vivo and in vitro models [19, 20]. Air pollution particles have been shown to translocate into and cross the placenta, which may occur by altering placental DNA methylation patterns that lead to changes in placental function and morphology [21, 22]. DNA methylation, one of the major epigenetic modifications, plays a vital role in the regulation of gene expression, genome stabilization, X-inactivation, genomic imprinting, and chromatin modification [23]. The aberrant methylation in the placenta is more likely to contribute to the onset of diseases such as RSA, preeclampsia, gestational diabetes, and preterm birth (PTB) [24–26]. It has been shown that DNA methylation may be affected in pregnant women exposed to air pollution. For example, women with exposure to traffic pollution during fetal development had a lower mean placental LINE-1 methylation level compared to those living farther from a major roadway and 7 CpG sites in the placenta were significantly associated with residential proximity to major roadways, which may be associated with lower fetal growth [27]. In addition, alterations in placental DNA methylation of the *LINE-1* and *HSD11B2* genes might be involved in PM10-induced reproductive and developmental toxicity [28]. Furthermore, NO_2_ exposure during early pregnancy influenced placental DNA methylation in preeclampsia and resulted in placental immaturity and sexual dimorphism [29]. In addition, the researcher found that the epigenetic modifiers such as *TET2* and *TET3* were upregulated in the placenta of patients with spontaneous abortion [30]. Genome-wide methylation sequencing of patients with RSA showed that *SGK1* methylation level in decidua tissue was significantly increased, which reduced cell proliferation and activity [31]. Moreover, *P53* methylation level is regulated by methyltransferase G9aMT and DNMT1 and hypomethylated in the decidua of RSA patients, which is negatively correlated with cell apoptosis and affects pregnancy maintenance [32]. Therefore, our primary hypothesis is that air pollution is associated with the risk of RSA. We secondary speculate that DNA methylation alterations may be involved in the biological mechanisms linking air pollution to RSA and could provide mechanistic clues to the pregnancy effects of exposure to air pollutants. Here, we performed bulk RNA-seq, reduced representation bisulfite sequencing (RRBS), and scRNA-seq on decidual tissues from individuals with induced abortion pregnancies and with RSA to explore the mechanisms of RSA and the association between RSA and air pollution.

## Results

### Genome-wide DNA methylation analysis of decidual tissues in RSA and controls

**Fig. 1:**
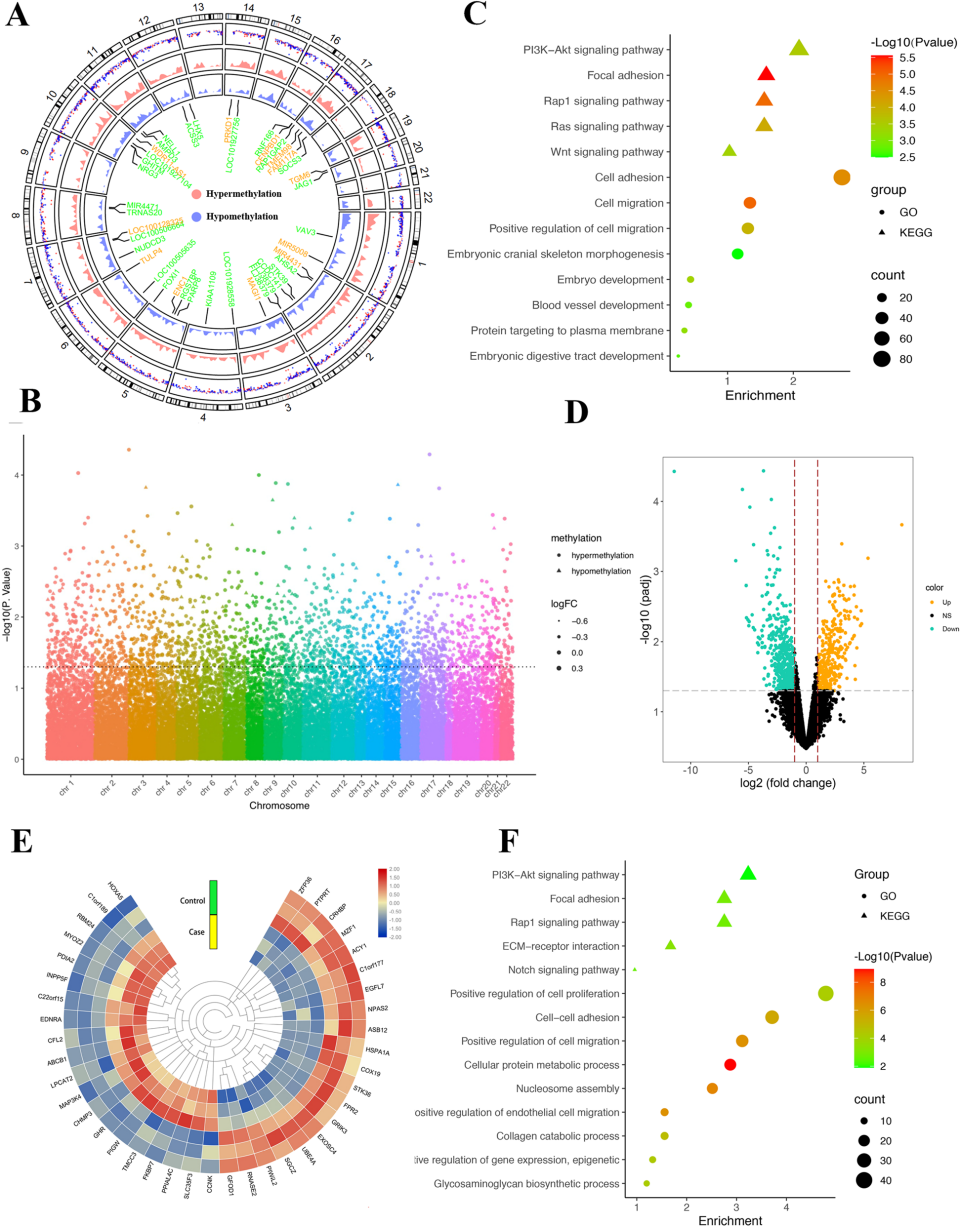
Differential DNA methylation distribution in decidual tissues. **A** Circular plot of different genes in the decidual samples. The two innermost circles represent the differential hypermethylation and hypomethylation frequencies. **B** Manhattan plot showing P value of each gene’s methylation level in decidua between RSA and controls. The horizontal line is the suggestive DNA methylation sequence significance threshold cutoff. **C** Enrichment analysis of DMGs. **D** Volcano plot for the DEGs. **E** Heatmap plot of the top 40 DEGs (20 upregulated DEGs and 20 downregulated DEGs). **F** Enrichment analysis of DEGs

To further understand the function of DNA methylation in RSA, we performed the RRBS in decidua samples from the patients with RSA (*n* = 3) and controls (*n* = 3). An average of 7.12 × 10^7^ sequencing data were obtained from the decidua RRBS library, with an average alignment rate of 87.07%. In addition, the average proportion of decidua tissue methylation in the control group and RSA case group was 46.17% and 45.27%, respectively. A total of 4133 differentially methylated regions (DMRs) targeting 3526 differentially methylated genes (DMGs) were identified (*P* < 0.05 and |*t*|> 2.5), of which 1944 DMRs targeting 1645 DMGs showed hypermethylation, whereas 2187 DMRs targeting 1881 DMGs were hypomethylated in RSA compared to the controls (Fig. 1A, Additional file 2: Table 2). DMRs were evenly distributed in the autosomes (Fig. 1B). GO analysis indicated an enrichment of DMGs that are implicated in the developmental processes, including embryonic cranial skeleton morphogenesis, embryo development, and embryonic digestive tract development (Fig. 1C). KEGG analysis indicated that these DMGs were mainly involved in Rap1, Ras, and PI3K-Akt signaling pathways (Fig. 1C). In total, abnormal DNA methylation is closely associated with RSA.

### Distinct DEGs of RNA-seq were identified in RSA and controls

In order to better explore the expression of related genes associated with RSA, we used bulk RNA-seq from three patients with RSA and three matched controls screened in RRBS. After deleting some unannotated genes whose names could not be obtained, 22,508 genes were obtained. A total of 928 differentially expressed genes (DEGs) were identified, including 380 downregulated DEGs and 548 upregulated DEGs. A volcano plot of the screened DEGs is shown in Fig. 1D. Furthermore, a heatmap of the top 40 DEGs (20 upregulated and 20 downregulated) is shown in Fig. 1E. These DEGs were mainly involved in cell adhesion, cellular metabolic processes, and cell migration (Fig. 1F). KEGG analysis showed that the 928 DEGs were associated with ECM–receptor interaction, focal adhesion, and the Rap1 signaling pathway (Fig. 1F).

### Single-cell expression profiling of DEGs identified in deciduae from RSA patients

**Fig. 2:**
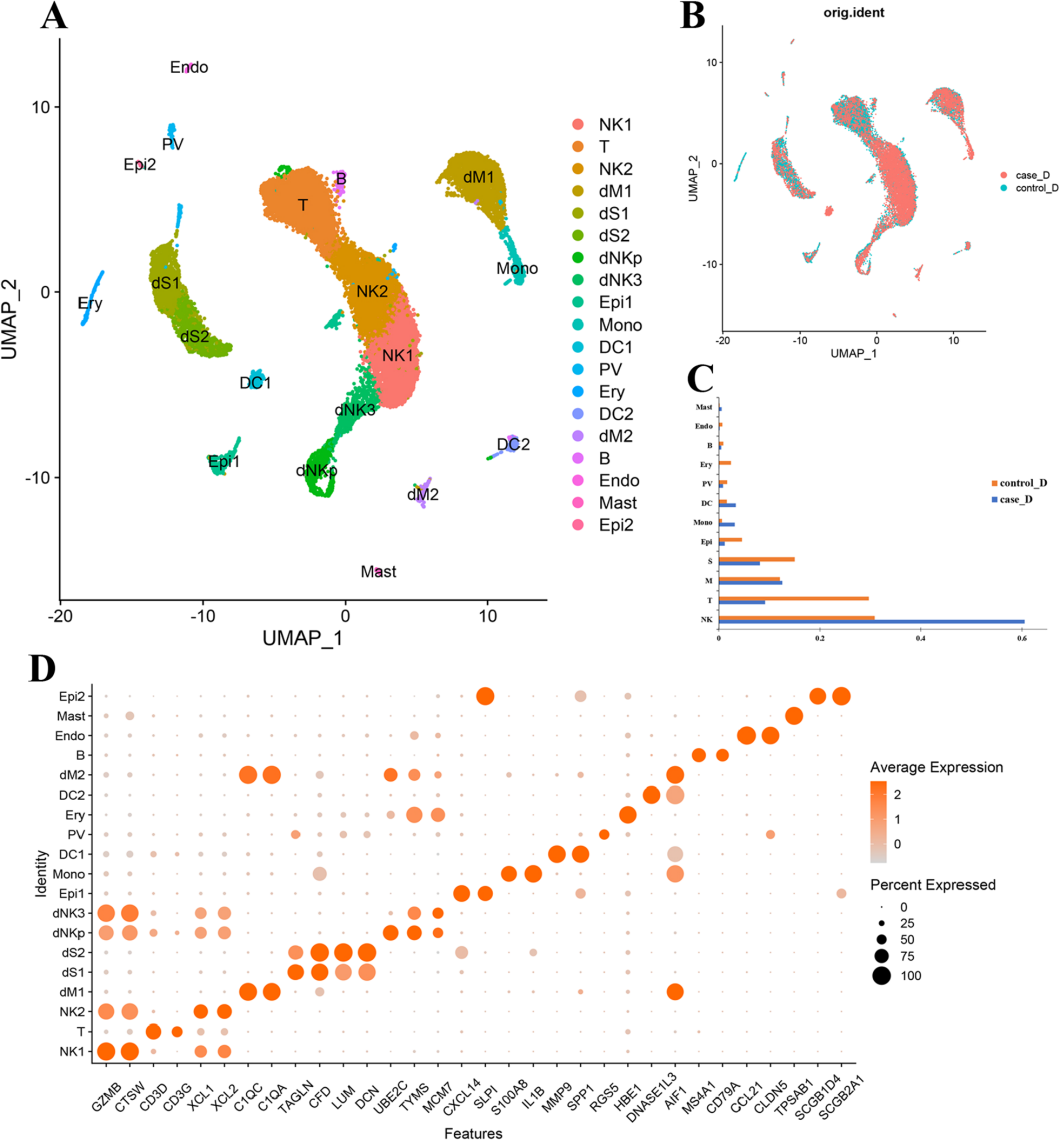
An atlas of decidual cells in patients with RSA. **A**, **B** A UMAP projection of all cells from one patient with RSA and one matched healthy control. Different colors indicate cell clusters. **C** Box plot highlights the contribution of two groups to each cell cluster. **D** Dot plot shows the expression of known cell markers

We obtained 19,416 high-quality cells in the final datasets for further analyses. Of these, 9377 cells originated from individuals with normal pregnancies and 10,039 cells originated from patients with RSA. Nineteen transcriptionally unique cell subsets were identified based on the expression of known marker genes and literature evidence (Fig. 2A, B, D). The three most abundant populations appeared to be decidual NK (dNK) cells, T cells, and decidual macrophages (dM) (Fig. 2C). Besides, we identified 604 DEGs compared with controls, comprising 235 downregulated DEGs and 369 upregulated DEGs in patients with RSA in scRNA-seq.

The dNK cells were clustered as dNK1, dNK2, and dNK progenitor cells (dNKp), which express the NK cell markers GZMB and CTSW. The T cells were annotated by specific genes such as CD3D and CD3G. The dM cells that express C1QA and C1QC were grouped into three major clusters, namely dM1, dM2, and dM progenitor (dMp) cells. Markers that distinguish the different decidual stromal (dS) cell populations identified two clusters that share expression of *TAGLN*, *CFD*, *LUM*, and *DCN*. In addition, a low abundance of epithelial glandular cells (Epi, expressed CXCL14 and SLPI), endothelial cells (Endo, annotated based on CCL21 and CLDN5 expression), mast cells (identified based on TPSAB1 and CPA3 expression), dendritic cells (DC, annotated based on DNASE1L3 and LGALS2 expression), and CD79A-marked B cells were identified (Fig. 2D). CellChat analysis showed that the status of signaling activation was different between RSA and normal pregnancies (Additional file 3: Fig. 1). Epi2 cells, for example, actively interacted with other cells in the normal decidua, whereas Epi2 cells in the RSA decidua showed no connection with other cells. Furthermore, the overall cell–cell interactions in RSA decidua were greatly elevated, which is consistent with earlier findings, indicating that our result is reliable [33].

### Molecular aspects and developmental trajectories of dNK and dM subtypes

Considering that dNK and macrophages are the most abundant leukocyte, we examined the cellular heterogeneity of dNK and macrophages in the decidua immune microenvironment. As shown in Additional file 4: Fig. 2A and B, the dNK1 cells preferentially expressed *GNLY*, *LAYN*, and *GZMB*, the dNK2 cells showed a high expression of the cytolytic enzymes *XCL1*, *MATK*, and *CCL5*, and the dNK3 subset is positive for expression of *GINS2* and *MCM5*. The dNKp cells strongly expressed the cell cycle-related genes *STMN1*, *TUBB*, *TYMS*, and *UBE2C*, suggesting that dNKp exhibited high proliferative activity (Additional file 4: Fig. 2C). As shown in Additional file 4: Fig. 2D, the dNKp cells that highly expressed cell cycle-associated genes were ordered at the root of the pseudotime trajectory, and sequentially followed by dNK1, dNK2, and dNK3.

We grouped dM cells into two subsets according to the marker genes (Additional file 5: Fig. 3A and 3B). Interestingly, we also observed that *UBE2C*, *TYMS*, and *TUBB* were highly expressed in dM2, which illustrates that dM2 is also associated with the cell cycle (Additional file 5: Fig. 3C). We further analyzed the developmental trajectory of dM cells using pseudotime analysis, and the dM2 subsets were ordered at the root of the pseudotime trajectory (Additional file 5: Fig. 3D). In summary, there was an imbalanced immune microenvironment at the maternal–fetal interface in patients with RSA, but the function of these cells remains to be further determined.

### Correlation of DMRs with genome-wide gene expressions and validation

**Fig. 3:**
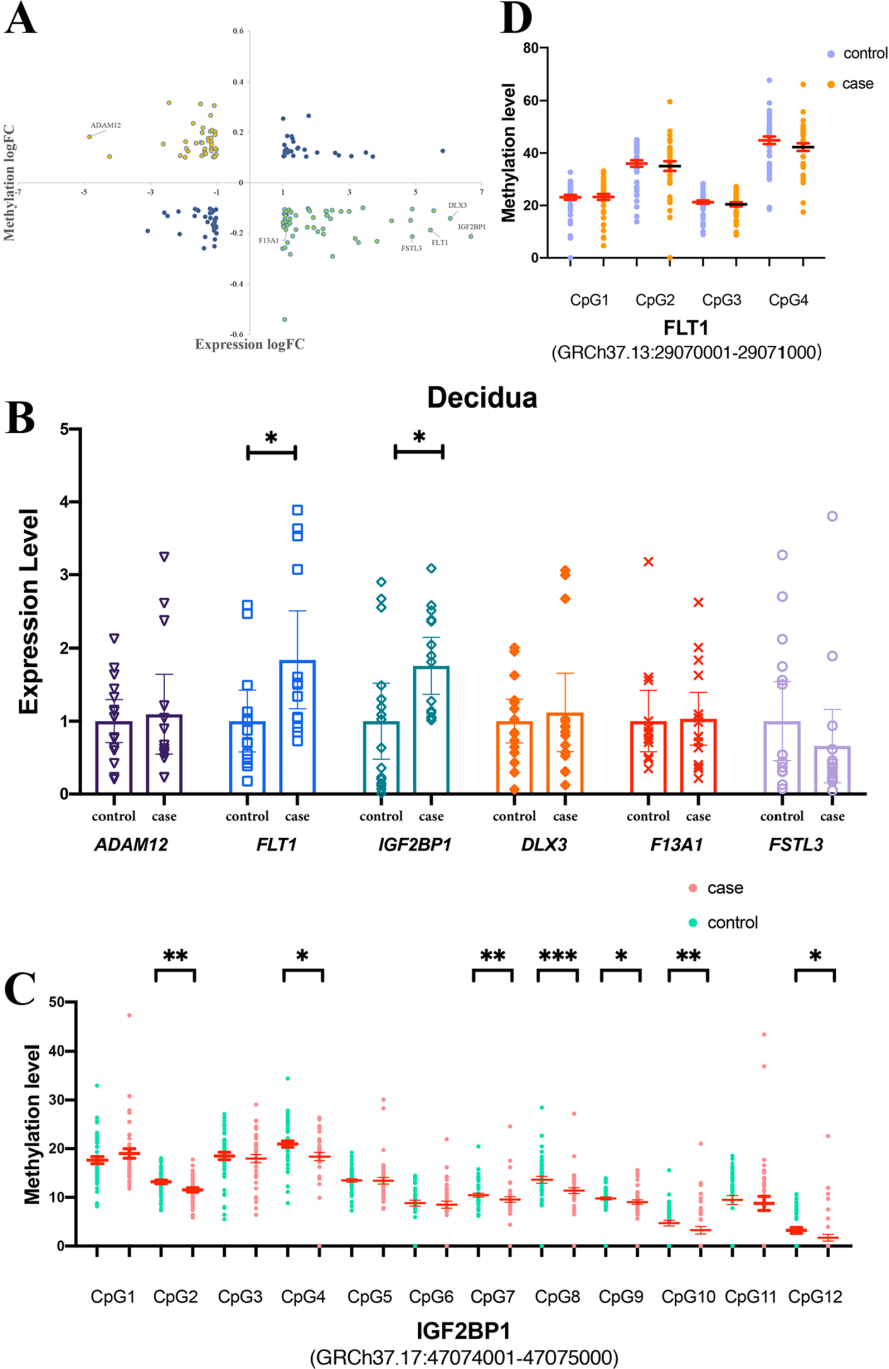
Integrated and validated analysis for gene expression profiling and DNA methylation. **A** Four-quadrant diagram shows DMRs and the expression of corresponding DEGs. Genes with yellow color are hypermethylated downregulated DEGs, and genes with green color are hypomethylated upregulated DEGs. **B** Dot plot of the relative expression level of screened six genes. *P < 0.05, **P < 0.01. The results of pyrosequencing of IGF2BP1 (**C**) and FLT1 (**D**)

To explore how methylation affects expression, we used an integrated analysis strategy. After integrating methylomes and transcriptomes, ninety-eight DEGs with abnormal methylation were identified, comprising fifty-five hypomethyl-upregulated DEGs and forty-three hypermethyl-downregulated DEGs (Fig. 3A). According to the literature and enrichment analysis, six genes (*ADAM12*, *FLT1*, *DLX3*, *IGF2BP1*, *F13A1*, and *FSTL3*) were screened and verified by qRT-PCR. The qRT-PCR validation was performed in 16 controls and 16 patients with RSA. As shown in Fig. 3B, the expression levels of *FLT1* and *IGF2BP1* in the decidua were significantly higher in patients with RSA than in controls. Subsequently, a pyrosequencing assay was performed on two genes (*FLT1* and *IGF2BP1*). We identified that DNA methylation levels of seven CpG sites in the *IGF2BP1* promoter region in the decidua were significantly different between the patients with RSA and controls (Fig. 3C, Additional file 1: Table 3), while no CpG sites in the *FLT1* promoter showed a statistical difference (Fig. 3D). Next, we performed receiver operating characteristic (ROC) curve analysis to examine the potential diagnostic value of methylation of *IGF2BP1* in distinguishing patients with RSA and controls, indicating that the methylation level of *IGF2BP1* has considerable potential as a diagnostic biomarker (Additional file 6: Fig. 4A). Interestingly, *IGF2BP1* was also observed in 369 upregulated DEGs of scRNA-seq and was highly expressed in dM1 and dNK1 (Additional file 6: Fig. 4B). Collectively, these findings illustrated the abnormal methylation and expression level of *IGF2BP1* may relate with RSA.

### The association between air pollution and RSA

We observed that the concentrations of these six air pollutants changed periodically, and air pollution was more serious in the cold period (Additional file 7: Fig. 5A and B). Among all the air pollutants, PM2.5 was highly positively correlated with PM10 (*r* = 0.907) and CO (*r* = 0.801) (Additional file 7: Fig. 5C); however, O_3_ was moderately negatively correlated with other air pollutants (*r* < 0.8). Because ozone formation is related to many factors, the relationship between ozone and RSA becomes very complicated due to these unknown factors [34]. Therefore, ozone was excluded from further analysis in this study. Detailed information about PM2.5, PM10, CO, NO_2_, SO_2_, and O_3_ is presented in Additional file 1: Table 4.

**Fig. 4:**
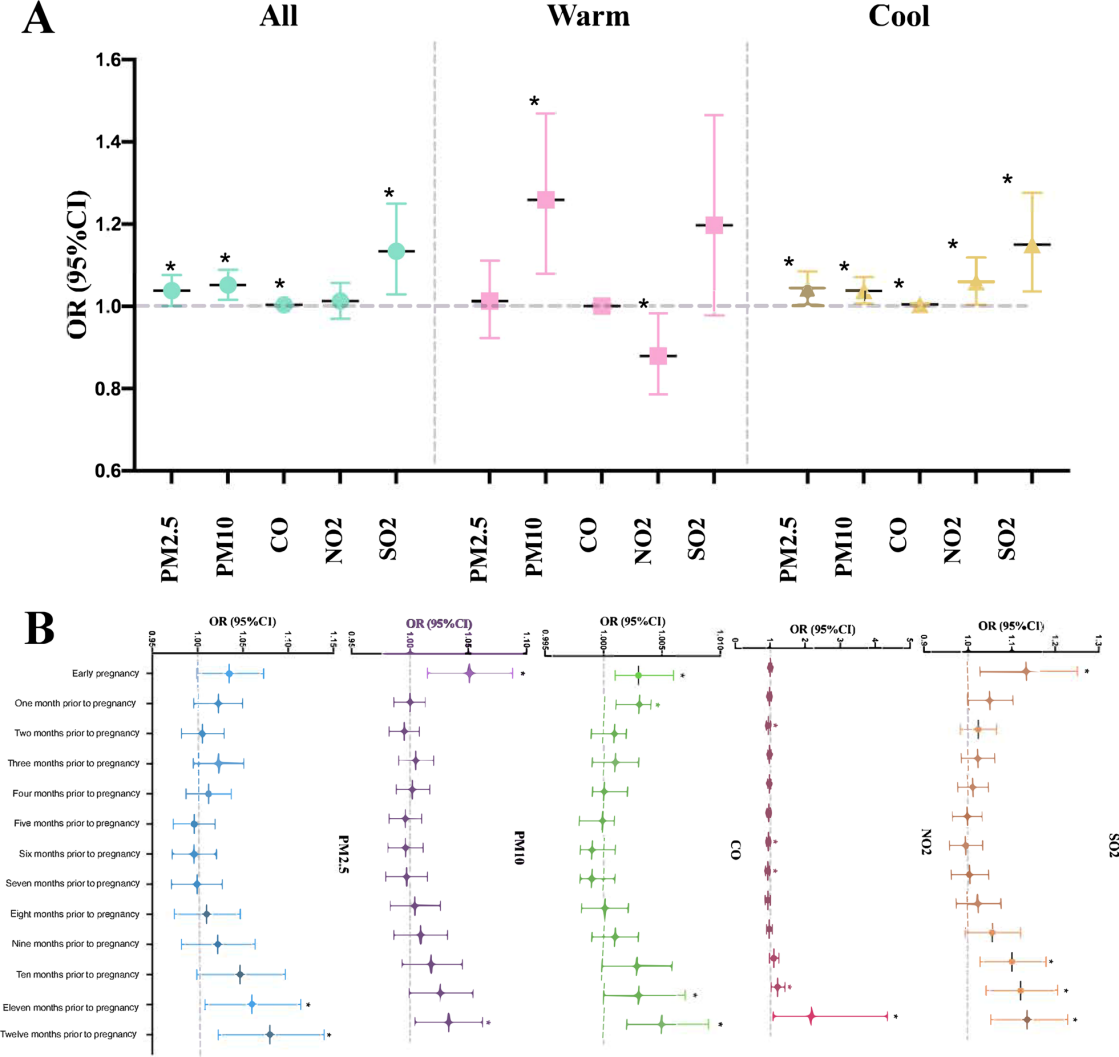
Relationship between air pollution and RSA. **A** Association between air pollution and RSA in different seasons. **B** Adjusted relative risks and their 95% CI for the association between RSA and each air pollutant from the first trimester to 1 year prior to pregnancy

As air pollution is a long-term health hazard, we analyzed the association between RSA and the monthly average air pollutants prior to pregnancy or in the early pregnancy (Table 1). As shown in Fig. 4B, we observed a significant positive association between maternal exposure to PM10 (OR 1.052, [95% CI 1.016, 1.089]), CO (OR 1.004, [95% CI 1.001, 1.006]), and SO_2_ (OR 1.134, [95% CI 1.029, 1.250]) in the early pregnancy and the risk of RSA. Furthermore, the mean estimated effects during the cool seasons on RSA were greater than those during the warm seasons, and all air pollutants in the cool seasons showed a significant positive association with the risk of RSA (Fig. 4A). Interestingly, we also found that the longer the exposure time in pre-pregnancy to the five air pollutants, the higher the risk of RSA, especially in the case of PM2.5 (OR 1.080, [95% CI 1.023, 1.140]), PM10 (OR 1.033, [95% CI 1.004, 1.062]), CO (OR 1.005, [95% CI 1.002, 1.009]), NO_2_ (OR 2.174, [95% CI 1.085, 4.356]), and SO_2_ (OR 1.137, [95% CI 1.053, 1.229]) in the year prior to pregnancy (Fig. 4B). In total, maternal exposure to air pollution was significantly associated with an increased risk of RSA.

**Table 1:** Association between different stages during or before pregnancy and exposure to six air pollutants

Air pollutants	Stage	Unadjusted		Adjusted	
		OR (95% CI)	*P* value	OR (95% CI)	*P* value
PM2.5	First trimester	1.038 (1.001, 1.076)	0.043	1.035 (0.999, 1.073)	0.060
	3 months before pregnancy	1.018 (0.992, 1.044)	0.181	1.023 (0.995, 1.051)	0.104
	6 months before pregnancy	0.992 (0.969, 1.016)	0.510	0.996 (0.972, 1.021)	0.758
	1 year before pregnancy	1.070 (1.016, 1.127)	0.010	1.080 (1.023, 1.140)	0.006
PM10	First trimester	1.052 (1.016, 1.089)	0.004	1.051 (1.015, 1.088)	0.005
	3 months before pregnancy	1.002 (0.988, 1.016)	0.805	1.005 (0.990, 1.020)	0.538
	6 months before pregnancy	0.993 (0.979, 1.008)	0.384	0.996 (0.981, 1.011)	0.608
	1 year before pregnancy	1.028 (1.001, 1.055)	0.045	1.033 (1.004, 1.062)	0.024
CO	First trimester	1.004 (1.001, 1.006)	0.003	1.003 (1.001, 1.006)	0.005
	3 months before pregnancy	1.001 (0.999, 1.002)	0.390	1.001 (0.999, 1.003)	0.254
	6 months before pregnancy	0.999 (0.998, 1.000)	0.120	0.999 (0.998, 1.001)	0.214
	1 year before pregnancy	1.005 (1.001, 1.008)	0.007	1.005 (1.002, 1.009)	0.005
NO_2_	First trimester	1.013 (0.970, 1.057)	0.564	1.007 (0.964, 1.052)	0.759
	3 months before pregnancy	0.972 (0.926, 1.020)	0.244	0.977 (0.930, 1.026)	0.353
	6 months before pregnancy	0.939 (0.896, 0.984)	0.009	0.943 (0.899, 0.990)	0.018
	1 year before pregnancy	2.231 (1.114, 4.469)	0.024	2.174 (1.085, 4.356)	0.029
SO_2_	First trimester	1.134 (1.029, 1.250)	0.011	1.135 (1.029, 1.251)	0.011
	3 months before pregnancy	1.017 (0.981, 1.054)	0.365	1.023 (0.985, 1.062)	0.242
	6 months before pregnancy	0.989 (0.954, 1.026)	0.571	0.995 (0.958, 1.034)	0.814
	1 year before pregnancy	1.127 (1.048, 1.213)	0.001	1.137 (1.053, 1.229)	0.001
O_3_	First trimester	0.980 (0.969, 0.992)	0.001	0.981 (0.970, 0.993)	0.002
	3 months before pregnancy	1.004 (0.990, 1.019)	0.554	1.002 (0.988, 1.017)	0.761
	6 months before pregnancy	1.011 (0.999, 1.023)	0.084	1.009 (0.997, 1.022)	0.157
	1 year before pregnancy	0.991 (0.941, 1.044)	0.729	0.982 (0.929, 1.037)	0.504

### Air pollution may cause recurrent abortions by affecting DNA methylation

**Fig. 5:**
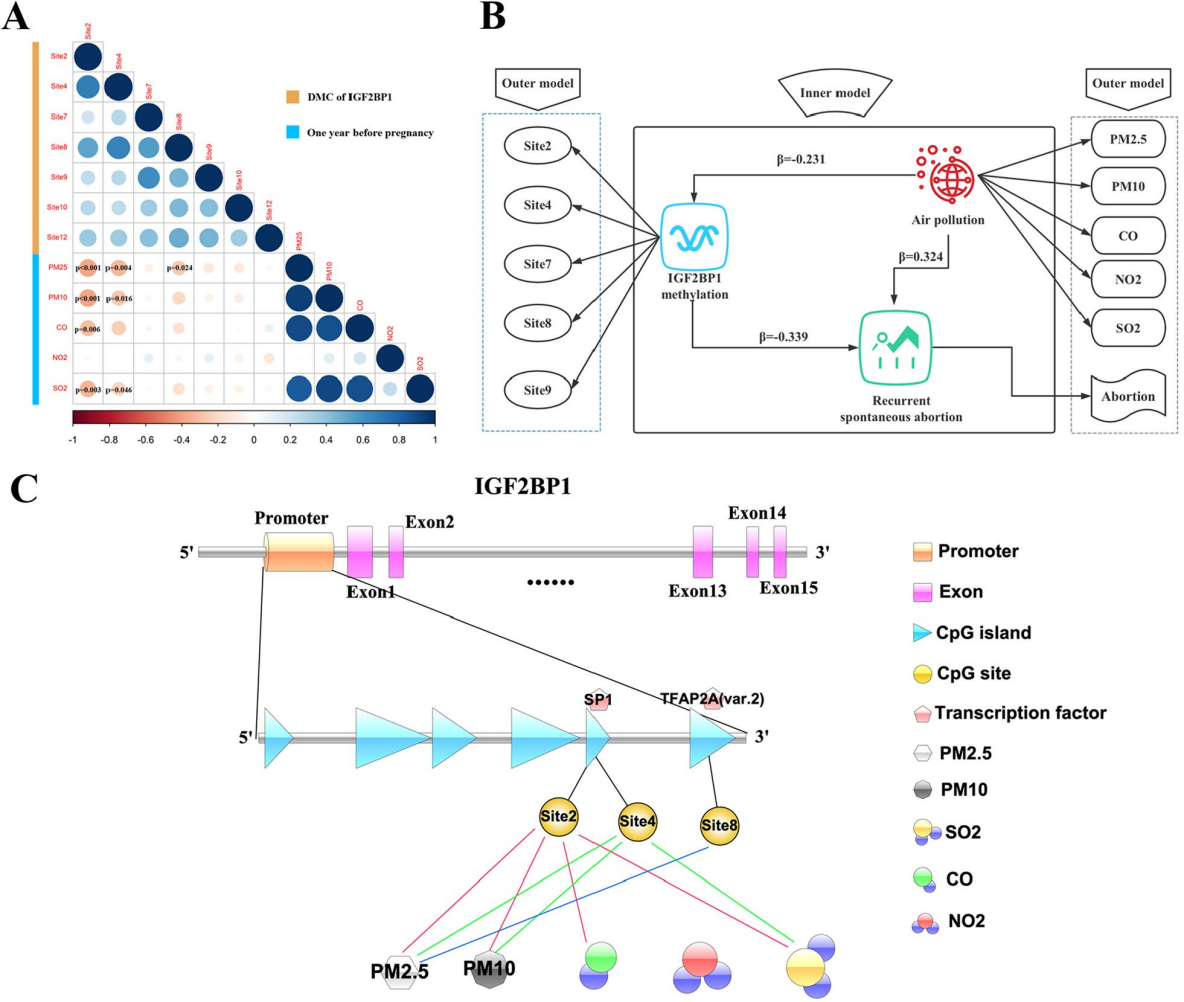
DNA methylation of IGF2BP1 promoter associated with air pollution. **A** Correlation heatmap plot shows the association between six DMC sites in IGF2BP1 and air pollution. **B** Path diagram indicating the conceptual model behind the relations among latent variables (inner model) and their manifest variables (outer model). **C** Schema of how the methylation level changes of IGF2BP1 promoter regions driven by air pollution

Six CpG islands were identified in *IGF2BP1* (Additional file 6: Fig. 4C), and 26 transcription factors (TFs) were predicted in the JASPAR database with predicted binding scores > 500 (*P* < 1 × 10^–5^) based on the CpG island location (Additional file 6: Fig. 4D). MAZ, ZNF148, KLF5, VEZF1, SP1, and TFAP2C were the most tightly bound TFs of the six islands. To uncover the relationship between alterations in DNA methylation and air pollution, we used the Spearman rank correlation to correlate the aforementioned seven significant differentially methylated CpG (DMC) sites of *IGF2BP1* and the concentration of the five air pollutants in the year preceding pregnancy that increased the risk of RSA. As shown in Fig. 5A, we observed that site2 and site4 of *IGF2BP1* showed a significant negative correlation with PM2.5, PM10, CO, or SO_2_. Additionally, site8 showed a significantly negative correlation with PM2.5. Since CpG site10 and site12 had a large number of 0 values and could not be normalized, we excluded these two sites from further analysis. Results of the associations between maternal air pollution exposure and the untransformed (site2 and site8) and ln-transformed (site4, site7, and site9) methylation level of *IGF2BP1* in the decidua using linear regression are shown in Table 2. After adjustment for the age of the mother, site2 *IGF2BP1* showed a significant negative correlation with PM2.5, PM10, CO, and SO2. Additionally, site4 was significantly negatively correlated with PM2.5 (Table 2, Fig. 5C). After constructing partial least squares path modeling (PLS-PM) diagrams of the latent variables of the *IGF2BP1* methylation sites and air pollution for RSA (Additional file 1: Table 5), air pollution showed a positive effect on RSA (*β* = 0.324, *P* < 0.001) but had a negative effect on *IGF2BP1* methylation (*β* = −0.231, *P* = 0.005). Besides, the association between *IGF2BP1* methylation and RSA was also negative (*β* = −0.339, *P* < 0.001). The overall association between air pollution and RSA can be partially explained by *IGF2BP1* methylation modulation (path coefficient = 0.402), which is calculated by summing up the indirect and direct effects (path coefficients = (−0.231) × (-−0.339)) and 0.324, respectively) (Fig. 5B). The obtained goodness of fit (GoF) was 0.411 (GoF > 0.3), which means the proposed model is globally fit. In addition, our results indicated that the total effect of air pollution on 24.2% RSA (ratio of an indirect to total effect) could be explained by the indirect effect of DNA methylation.

**Table 2:** Linear regression of maternal air pollution exposure and *IGF2BP1* methylation in the decidua

Air pollutants	CpG site	Unadjusted		Adjusted	
		OR (95% CI)	*P* value	OR (95% CI)	*P* value
PM2.5	site2	− 0.099 (− 0.16, − 0.038)	0.002	− 0.100 (− 0.161, − 0.038)	0.002
	site4	− 0.122 (− 0.245, 0.001)	0.051	− 0.129 (− 0.252, − 0.005)	0.042
	site7	− 0.022 (− 0.083, 0.038)	0.465	− 0.021 (− 0.082, 0.041)	0.500
	site8	− 0.063 (− 0.156, 0.030)	0.182	− 0.066 (− 0.159, 0.028)	0.169
	site9	− 0.046 (− 0.103, 0.011)	0.110	− 0.049 (− 0.107, 0.008)	0.091
PM10	site2	− 0.054 (− 0.087, − 0.02)	0.002	− 0.054 (− 0.088, − 0.020)	0.002
	site4	− 0.062 (− 0.130, 0.005)	0.071	− 0.067 (− 0.135, 0.001)	0.054
	site7	− 0.012 (− 0.045, 0.021)	0.472	− 0.011 (− 0.045, 0.023)	0.516
	site8	− 0.031 (− 0.082, 0.020)	0.225	− 0.033 (− 0.085, 0.018)	0.203
	site9	− 0.022 (− 0.053, 0.010)	0.170	− 0.024 (− 0.056, 0.008)	0.134
CO	site2	− 0.006 (− 0.010, − 0.002)	0.004	− 0.006 (− 0.010, − 0.002)	0.004
	site4	− 0.007 (− 0.015, 0.001)	0.073	− 0.007 (− 0.015, 0.000)	0.063
	site7	− 0.001 (− 0.005, 0.003)	0.616	− 0.001 (− 0.005, 0.003)	0.650
	site8	− 0.004 (− 0.010, 0.002)	0.222	− 0.004 (− 0.010, 0.002)	0.211
	site9	− 0.003 (− 0.007, 0.000)	0.082	− 0.003 (− 0.007, 0.000)	0.070
NO_2_	site2	− 0.119 (− 0.314, 0.075)	0.226	− 0.122 (− 0.318, 0.074)	0.220
	site4	− 0.237 (− 0.614, 0.141)	0.215	− 0.228 (− 0.608, 0.152)	0.236
	site7	− 0.006 (− 0.190, 0.178)	0.947	− 0.010 (− 0.196, 0.176)	0.916
	site8	− 0.090 (− 0.373, 0.194)	0.530	− 0.086 (− 0.372, 0.200)	0.550
	site9	− 0.149 (− 0.321, 0.023)	0.089	− 0.145 (− 0.318, 0.029)	0.101
SO_2_	site2	− 0.102 (− 0.174, − 0.030)	0.006	− 0.102 (− 0.174, − 0.030)	0.006
	site4	− 0.141 (− 0.284, 0.002)	0.054	− 0.143 (− 0.286, 0.001)	0.051
	site7	− 0.013 (− 0.083, 0.058)	0.725	− 0.012 (− 0.083, 0.059)	0.740
	site8	− 0.069 (− 0.177, 0.039)	0.209	− 0.070 (− 0.178, 0.039)	0.207
	site9	− 0.064 (− 0.130, 0.002)	0.057	− 0.065 (− 0.131, 0.001)	0.054

## Discussion

In this study, we integrated the results of RNA-seq and RRBS and obtained 98 DEGs with aberrant methylation in RSA decidual tissue, and most DEGs were involved in pathways associated with embryo implantation or development. Among these DEGs, the *IGF2BP1* gene was significantly expressed in patients with RSA and several CpG sites in the IGF2BP1 promoter regions showed significant hypomethylation levels compared with healthy controls. In addition, we observed an imbalance in the feto-maternal immune microenvironment of patients with RSA, according to the results of scRNA-seq. Furthermore, exposure to air pollution 1 year prior to pregnancy and in early pregnancy was positively related to a high risk of RSA. Thus, we hypothesized that there is a potential relationship among DNA methylation, air pollution, and RSA. PLS path modeling showed that air pollution exposure could affect the aberrant methylation of the *IGF2BP1* promoter and contribute to RSA developmental processes.

Previous studies have reported that dysregulation of DNA methylation may play a role in RSA [31, 35]. Yu et al. reported the promoter of *CREB5* was hypomethylated in RSA decidual tissues and it altered trophoblast cell functions [36]. We identified 98 DEGs with abnormal methylation by integrating RNA-seq and RRBS data. Furthermore, *IGF2BP1* and *FLT1* were significantly expressed in patients with RSA. *FLT1* is predominantly expressed in the mammalian placenta and forcibly expressed in the placenta during early pregnancy loss [37]. Moreover, several studies have indicated that *FLT1* and its alternative splicing *sFLT1* were expected to be biomarkers or therapeutic targets of adverse pregnancy [37–39]. No significant CpG sites were found in the DMRs of the *FLT1* promoter. A possible reason is that DNA methylation may not be related to FLT1 expression, which may be regulated by other elements or factors, such as transcription factors and noncoding RNAs.

Another gene, *IGF2BP1*, is enriched in human trophectoderm and placental trophoblast cells and involved in pregnancy establishment and maintenance [40]. Recent studies revealed that the association between *IGF2BP1* and target mRNAs is enhanced by N6-methyladenosine (m6A) modification of target transcripts, indicating that it is a new m6A reader that protects m6A-modified mRNA from degradation and promotes mRNA translation [41]. m6A is the most abundant RNA modification and plays a vital role in placental and embryonic development [42, 43]. At the maternal–fetal interface of women with spontaneous abortion, m6A itself is abnormally accumulated and correlated with the downregulated RNA demethylase *FTO* [44]. Shisu et al. reported that *IGF2BP1* was expressed at significantly higher levels in the uterus of patients with RSA [43]. Moreover, the seven differentially methylated CpG sites we detected were located on two different CpG islands, and 26 TFs involved in transcriptional regulation were predicted to bind to these CpG islands, which affected the expression of *IGF2BP1*. Based on the above research results, we speculated that the hypomethylation level in *IGF2BP1* promoter affected its expression, and the upregulated *IGF2BP1* might accumulate m6A to malfunction in the placenta, resulting in an increased risk of RSA.

The whole decidua represents an extremely complex mixture of cell types and estimates of gene expression in mixed samples are inherently inaccurate because it is difficult to adjust for differences in cell composition [45]. At the maternal–fetal interface, decidual NK cells and macrophages account for a large number of decidual leukocytes during early pregnancy. In patients with RSA, the pregnancy-promoting functions of dNK are dysregulated in several ways, such as increased concentrations and greater cytotoxicity of dNK as well as dysregulated angiogenesis [46]. Decidual macrophages can be differentiated into classically activated macrophages (M1) and alternatively activated macrophages (M2). M1 macrophages were abundant in RSA decidual tissues [47] and increased expression of CD86, suggesting that the dM regulatory capacity of Tregs is decreased in women with RSA [48]. These results suggest an imbalance in the feto-maternal immune microenvironment of patients with RSA.

Several studies have reported that exposure to air pollution during pregnancy increases the risk of spontaneous abortion [5, 49, 50]. Gaskins et al. reported that 1-year and 2-year exposures to PM2.5–10 prior to pregnancy were associated with a higher risk of spontaneous abortion [6]. In the present study, five air pollutants (PM2.5, PM10, CO, NO_2_, and SO_2_) were positively associated with the risk of RSA in the early pregnancy, suggesting that short-term exposure to poor air quality during pregnancy increases the likelihood of miscarriages. Furthermore, this trend is more obvious with long-term exposure before pregnancy. We focus on an exposure window spanning 12 months before pregnancy because we thought it was most relevant in subsequent placental development [6]. Based on our results, we hypothesized that long-term exposure to air pollution before pregnancy may affect the intrauterine environment for placenta formation, including endocrine disruptions and endometrial inflammation, thereby increasing the risk of RSA.

It is widely known that the effects of air on health outcomes might be modified by the seasons, and air pollutant emissions increased markedly during the winter months [51, 52]. For example, short-term NO_2_ exposure is significantly associated with an increased risk of RSA, and the effect of NO_2_ exposure is more pronounced in the cold season [53]. Our research is consistent with these findings that the associations between exposure to air pollutants and RSA events varied significantly with seasons and were especially pronounced in cool seasons. The reasons for the seasonal differences can be complex. The possible causes may be that heating is provided in various regions of Tianjin in cool seasons, which increases the emission of air pollutants, while there are more heavy rains in warm seasons to clean up ambient pollution [53].

Multiple mechanisms of action may be involved in abortion induced by air pollution, such as perturbations in oxidative stress, systemic and placental inflammation, endothelial dysfunction, DNA damage, and/or methylation, and these warrant further investigation [54, 55]. Increased exposure to ambient air pollution during pregnancy is associated with global loss of methylation in the placenta [28, 56]. Janssen et al. reported placental global DNA methylation was inversely associated with PM2.5 exposures during the whole pregnancy and relatively decreased by 2.19% for each 5 μg/m^3^ increase in exposure to PM2.5 [57]. Moreover, they observed only exposure to PM2.5 during the first trimester was significantly associated with lower global DNA methylation [57]. By analyzing the correlation between seven CpG sites and air pollutants, we found that three CpG sites of *IGF2BP1* showed a significant negative association with PM2.5, PM10, CO, or SO_2_. In addition, to test for associations in a network of causal relationships, we performed PLS-PM, which is applicable to small sample sizes and overcomes statistical limitations by combining information from multiple variables rather than assessing them one by one [58, 59]. Nahid et al. applied PLS-PM and indicated that 15% of the effect of air pollution on the risk of adult-onset asthma was mediated through immune system markers [60]. Our study suggested an association of air pollution and *IGF2BP1* methylation with RSA, and the model showed that IGF2BP1 methylation might be a 24.5% mediating effect of air pollution on RSA. We hypothesize that exposure to air pollution in the 12 months prior to pregnancy affects the endometrial environment and affects placental development. Placental development is closely related to DNA methylation, and abnormal DNA methylation changes gene expression, which may lead to abortion.

This study had several limitations. The first may be the small size of the study population in RRBS and RNA-seq. We prioritized high-quality DNA and RNA samples from previously collected samples for RRBS and RNA-seq analysis due to the restrictions of early experimental settings, in order to get methylation and expression data as early as feasible. The characteristics of the six subjects used for the RNA-seq and RRBS analysis, age, BMI, and gestational age were matched. Then, to get more accurate results, a significant sample of the studied genes was validated. Most importantly, the qPCR and pyrosequencing validation results of 77 samples in our study were consistent with the prediction of RNA-seq and RRBS. Therefore, we did not further increase the number of samples for RNA-seq and RRBS. Although the sample size is small, this may not affect our conclusions. Second, we identified aberrant expression and methylation of *IGF2BP1* in RSA, and the methylation level in its promoter was closely related to air pollution. This result lacks in vivo and in vitro experimental validation, which should be addressed in further studies. Third, we only studied decidual tissue and RSA is a multifactorial and multi-organ related disease, which could require integrated studies of multiple tissues from patients with RSA. Fourth, the indoor environment of work and residence was not detected, and the air pollution concentrations indoor and outdoor may be different, which may underestimate the impact of air pollution on abortion, but this method is the most widely used and practicable way to assess air pollution exposure [61].

There are many factors affecting DNA methylation, including different gestational age, lifestyle, diet, maternal–fetal immune tolerance microenvironment, and other factors. Wheater et al. reported that DNA methylation pattern is associated with differentially gestational age [62]. Differential DNA methylation may affect gestational age at birth through cell–cell membrane adhesion molecules [63]. In addition, the researchers also reported that maternal adverse lifestyle may affect the intrauterine environment by altering DNA methylation, resulting in adverse pregnancy outcomes such as abortion [64, 65]. Therefore, the difference in DNA methylation between RSA patients and normal controls is not only related to air pollution, but also may be related to different gestational age, adverse lifestyle, and other factors.

## Conclusion

In summary, the current study of patients with RSA revealed abnormal gene expression, epigenetic modifications, and immune atlas, which contribute to the pathogenesis of RSA. In addition, increased PM2.5, PM10, NO_2_, SO_2_, and CO were positively associated with the risk of RSA, especially in cool seasons. Importantly, we identified and verified that *IGF2BP1* was significantly upregulated in RSA decidua, and the methylation levels of seven CpG sites were significantly decreased, of which five CpG sites were closely correlated with air pollution. Furthermore, the current study may provide supportive evidence for a 24.5% mediating effect of *IGF2BP1* methylation in the association between RSA and air pollutants. Therefore, we provide a new direction for RSA research.

## Materials and methods

### Study population and sample collection

From November 1, 2014, to December 31, 2018, a total of 86 patients with RSA and 128 normal women in early pregnancy (6–12 weeks of gestation) were recruited from the gynecology and obstetrics outpatient department of the Second Hospital of Tianjin Medical University. After excluding individuals with infection, embryo chromosomal abnormalities, endocrine abnormalities, hypertension, anatomic abnormalities, antiphospholipid syndrome, or other known risk factors, 77 participants including 31 patients with unexplained RSA and 46 controls were included for further analyses. The clinical data for women with and without RSA are presented in Table 3. At the time of dilation and curettage, we separated decidual tissue from the products of conception. The decidual tissues were flash-frozen in liquid nitrogen and stored at − 80 °C for subsequent studies. The study was approved by the Ethics Committee of Shanghai Institute of Planned Parenthood Research, and written informed consent was obtained from each participant.

**Table 3:** Characteristic distribution of the subjects

Characteristics	Medical abortion samples (controls, *n* = 46)	Spontaneous abortion samples (cases, *n* = 31)	*P* value
Age (years)	29.52 ± 6.62	31.48 ± 4.71	0.133
Reproductive history (parity)	0.80 ± 0.75	0.16 ± 0.37	< 0.001
Abortion history	1.18 ± 0.86	3.10 ± 1.80	< 0.001
Gravidity	2.80 ± 1.56	3.35 ± 1.80	0.151
Menolipsis days	52.45 ± 8.47	61.48 ± 9.80	< 0.001

Values are represented as median ± SD

### DNA isolation and RRBS

Genomic DNA from the decidua was extracted and purified using the Qiagen DNeasy Kit (Qiagen) according to the manufacturer’s instructions. DNA from three patients with RSA and three matched controls were randomly selected for sequencing. Genomic DNA was digested with the MspI restriction enzyme, and digests of the desired size (40–220 bp DNA fragments) were extracted using NuSieve Gels. We used the FastQC application (http://www.bioinformatics.babraham.ac.uk/projects/fastqc) to evaluate the quality of sequencing data. The sequenced reads were mapped against the complete human reference genome GRCh37/h19 using Bismark software (https://www.bioinformatics.babraham.ac.uk/projects/bismark/) and the proportion of cytisine and thymine bases was calculated at CG positions among the bisulfite sequencing reads aligned to the reference sequencing as the methylation level. Data processing and analyses were performed using *methylKit* package, a package is used to descript the methylation statistics of all samples and find differentially methylated regions from sorted Bismark-aligned BAM files in software R (Version 4.1.0). A logistic regression test will be applied to compare the fraction of methylated Cs across the patients with RSA and the controls [66]. In *methylKit*, *tileMethylCounts* function was used to discover de novo differentially methylated genes (DMRs). DMRs were defined as a sliding window with a default window size of 1000 bp. Specifically, statistical tests for differential methylation at each region were performed by function “calculateDiffMeth” with default parameters, which output was then processed using function “getMethylDiff” to call DMRs by comparing the RSA group with the control group. Then, *ChIPseeker* was used to annotate the differentially methylated regions.

### RNA isolation and RNA-seq

Total RNA from decidual tissue was extracted using TRIzol reagent (Life Technologies). The six participants used for RRBS are the same as the six participants used for RNA-seq. RNA-seq data were normalized and analyzed using the *limma* package to identify DEGs. The *limma* package evaluated differential expression analysis by fitting a linear equation to the expression level of each gene using a generalized linear model [67]. Genes with adjusted *P* < 0.05, and | log2FC |≥ 1 were selected for further analyses.

### Isolation of decidual cells and single-cell RNA-seq

Decidual tissues were minced into small pieces and digested with an enzyme cocktail containing collagenase V and trypsin. Following diluting in Dulbecco’s modified eagle medium (DMEM), the cell suspensions were filtered through a 70um cell strainer and centrifuged at 500 g for 10 min. The cell pellets were resuspended in 1 mL DMEM containing 10% FBS and loaded onto a discontinuous Percoll to obtain the single-cell suspension. Cell viability was assessed using Trypan blue exclusion. The cells were counted and diluted at a concentration of 1000 cells/uL, aiming for an estimated 10,000 cells per library.

The cell suspensions and barcoded beads were loaded onto a custom-built microfluidics single-cell chip along with a reverse transcription master mix, and then monodispersed droplets were generated. After amplifying using a TouchTM Thermal Cycler (Bio-Rad), the cDNA molecules were tagmented and amplified using the Nextera XT DNA Sample Prep Kit (Illumina). Single-cell libraries were sequenced on the Illumina HiSeq X Ten platform.

### Single‐cell RNA‐sequencing data analysis

We converted the obtained matrix into a Seurat object using the Seurat package (Version 4.0.2) and integrated the single-cell data using the *Harmony* package [68]. Finally, the RunPCA and RunTSNE functions were used to linearly scale the expression data and nonlinearly scale dimensionality reduction. In scRNA-seq, genes with *P* < 0.05 were identified as DEGs from the comparison of the patients with RSA and controls.

A single-cell trajectory was constructed using a matrix of cells and their gene expression profiles using the *monocle* package (Version 2.20.0). We also applied the *CellChat* algorithm to predict the major signaling inputs and outputs of the cells [69].

### Enrichment analysis

The Database for Annotation, Visualization, and Integrated Discovery (DAVID, https://david.ncifcrf.gov/home.jsp) was used to perform Gene Ontology (GO) annotations and Kyoto Encyclopedia of Genes and Genomes (KEGG) pathways analysis. The threshold of significance was defined by *P* value < 0.05.

### Exposure assessment

Assessed ambient exposure data on air pollutants were routinely recorded by the National Science & Technology Infrastructure of China (http://henu.geodata.cn) from November 1, 2013, to December 31, 2018; they included six major pollutants: O_3_, SO_2_, NO_2_, CO, PM2.5, and PM10. The average of 24-h data obtained from 27 monitors within the whole city of Tianjin was taken as the daily concentrations for each air pollutant and the 27 monitors covered the air environment outside the work and home addresses of all participants. According to the date of last menstruation and the date of surgery, the timing windows for environmental factor exposure were divided into prenatal and postnatal periods. The 12 months before the last menstrual period were named as pre-pregnancy, and the period between the last menstrual period and the operation date was defined as early pregnancy.

### Real-time qPCR analysis

RNA samples (1 µg) were converted to cDNA using the cDNA Synthesis Kit (Thermo Scientific) according to the manufacturer’s instructions. DEGs were validated by qRT-PCR in a LightCycler 480 (Roche) along with the *β-actin* as an internal control. Statistical analysis was performed using a *t* test for the comparison of **ΔΔ**Ct values. The primers used for validation are listed in Additional file 1: Table 1.

### Pyrosequencing analysis

The sequences of primers used for pyrosequencing are listed in Additional file 1: Table 1. After amplification using the HiFi PCR Kit (KAPA), the PCR products were combined with the reaction binding beads and placed in a pyrosequencing detector (PyroMark q96 ID, Qiagen) for the reaction. Sequencing data were analyzed using the Pyro Q-CpG software.

### Statistical analyses

For the analysis of sequencing profiling data, statistical analysis of all sequencing data was performed using R (Version 4.1.0). Due to the skewed methylation levels of *IGF2BP1* and *FLT1*, the Kolmogorov–Smirnov test was used to examine the differences in methylation levels between patients with RSA and controls. Hypomethyl-upregulated DEGs and hypermethyl-downregulated DEGs were derived by overlapping DEGs and putative DMR targets [70]. The average air pollutant exposures were calculated for each participant for each month of the 1 year prior to conception (from 1 month prior to pregnancy to 12 months prior to pregnancy) and early pregnancy was used for subsequent analysis. A logistic regression model adjusting for the age of the mother was used to estimate the odds ratio (OR) and 95% confidence intervals (CI) for increased pregnancy loss with exposure to air pollutants of 77 participants enrolled. We also performed a stratified analysis to investigate the potential health effects modified by season (warm: April to September; cool: October to March). In our study, we assigned 43 subjects including 11 patients with RSA and 32 controls to the warm season and 34 subjects including 20 patients with RSA and 14 controls to the cool season based on the date of surgery. The correlations between methylation levels of *IGF2BP1* and air pollutants prior to 1 year to pregnancy were assessed using Spearman correlation coefficients. Methylation levels of *IGF2BP1* at differentially methylated CpG dinucleotide (site4, site7, and site9) were ln-transformed to approximate normal distributions. Additionally, we constructed a linear regression model on each CpG dinucleotide of IGF2BP1 adjusting for the age of the mother in 77 participants to evaluate the associations between methylation and air pollution. PLS-PM was carried out to evaluate the potential mediating effect of the methylation level of the *IGF2BP1* promoter on the association between air pollution and RSA in 77 participants using SmartPLS (version 3.3.3). In PLS-PM, latent variables are used to consider all relationships between explanatory and manifest variables [71]. To test the overall model fit for PLS-SEM, GoF was calculated to identify the PLS model globally [72]. The Fisher’s exact test, Spearman correlation analysis, *t* test, Mann–Whitney *U* test, and chi-square test were performed using the SPSS software (Version 25.0).

## Supplementary Information

**Additional file 1**: Table 1: Primers for genes validated using qRT-PCR and pyrosequencing sequencing primers. Table 3. The methylation levels of CpG sites in the promoter regions of FLT1 and IGF2BP1 were detected by pyrosequencing. Table 4: Descriptive indices of air pollutants and climate factors in the city Tianjin from January 1, 2017 to December 31, 2018 (reported as per day). Table 5: PLS-PM analysis for the relationships between latent variables.

**Additional file 2**: Table 2. Total of DMR between patients with RSA and controls detected through RRBS.

**Additional file 3**: Fig. 1. Cell communication analysis between 14 cluster cells. Circle plot shows the number of interactions and interaction weights/strength of 14 cluster cells between case and control.

**Additional file 4**: Fig. 2. Molecular details and subclusters of dNKs were revealed by scRNA-seq. (A) A UMAP projection of the dNKs from one RSA patient and one matched healthy control. Different colors indicate cell clusters. (B) Dot plot shows the expression of marker genes for each subcluster of dNK. (C) Dot plot shows the expression of cell cycle-related genes. (D) Developmental trajectories of dNK subsets, cells colored by conditions of trajectories state, groups, subclusters, and pseudotime.

**Additional file 5**: Fig. 3. Molecular details and subclusters of dM were revealed by scRNA-seq. (A) A UMAP projection of the dM from three RSA patients and three matched healthy controls. Different colors indicate cell clusters. (B) Dot plot shows the expression of marker genes for each subcluster of dM. (C) Dot plot shows the expression of cell cycle-related genes. (D) Developmental trajectories of dM subsets, cells colored by conditions of trajectories state, groups, subclusters, and pseudotime.

**Additional file 6**: Fig. 4. Analysis of IGF2BP1 expression and its promoter region. (A) ROC analysis of methylation of IGF2BP1 in patient with RSA and controls. (B) scRNA-seq analysis of the expression of IGF2BP1 in 14 cell subsets. (C) Distribution of CpG island in the IGF2BP1 promoter region. (D) Prediction of transcription factors binding to each CpG island.

**Additional file 7**: Fig. 5. The relationship between air pollution-related genes and RSA. (A) and (B) Monthly air pollutant concentration curve from 2014 to 2018. (C) Correlation heatmap plot shows the association between six air pollutants.

## Data Availability

Datasets generated during and/or analyzed during the current study are not publicly available but are available from the corresponding author on reasonable request.

## Abbreviations

DEGs: Differentially expressed genes
DMG: Differentially methylated gene
DMRs: Differentially methylated regions
m6A: N6-methyladenosine
PLS-PM: Partial least squares path modeling
PM: Particulate matter
PTB: Preterm birth
RSA: Recurrent spontaneous abortion
RRBS: Reduced representation bisulfite sequencing
scRNA-seq: Single-cell RNA sequencing
TF: Transcription factor
NO_2_: Nitrogen oxide
CO: Carbon monoxide
SO_2_: Sulfur dioxide
O_3_: Ozone
GoF: Goodness of fit (GoF)
