# Supplementary material for: Integrated single-cell RNA-seq and DNA methylation reveal the effects of air pollution in patients with recurrent spontaneous abortion
Source: Clin Epigenetics. 2022 Aug 23;14:105. doi: 10.1186/s13148-022-01327-2 (PMC9400245; doi:10.1186/s13148-022-01327-2)
Supplement: Supplementary file 1 — Additional file 1: Table 1: Primers for genes validated using qRT-PCR and pyrosequencing sequencing primers. Table 3. The methylation levels of CpG sites in the promoter regions of FLT1 and IGF2BP1 were detected by pyrosequencing. Table 4: Descriptive indices of air pollutants and climate factors in the city Tianjin from January 1, 2017 to December 31, 2018 (reported as per day). Table 5: PLS-PM analysis for the relationships between latent variables. [file 13148_2022_1327_MOESM1_ESM.docx]

Supplementary Table 1. Primers for genes validated using qRT-PCR and pyrosequencing primers

| Genes | Primer | Sequences |
| --- | --- | --- |
| *qRT-PCR primers* |  |  |
| IGF2BP1 | IGF2BP1 F | GCAAGACCTTACCCTTTACAACC |
|  | IGF2BP1 R | GGCAGCCACCTAATTCTCATAG |
| FTL1 | FTL1 F | CGTAAGGCGACGAATTGACC |
|  | FTL1 R | TGATGGTCCACTCCTTACACGAC |
| DLX3 | DLX3 F | TACAAGAACGGGGAGGTGCC |
|  | DLX3 R | GGGTGATGGTGGTGAGTTGC |
| ADAM12 | ADAM12 F | TCCAGCAACTCCTGTGACCTC |
|  | ADAM12 R | GCAGTAGCCGTCCACATCCT |
| F13A1 | F13A1F | GGTTCAAGCCATCAAGCACG |
|  | F13A1 R | TCCACCACATGAGTGCCATC |
| FSTL3 | FSTL3 F | CCTGGTGCTCCAGACTGATGT |
|  | FSTL3 R | CAAGAAGCCGAGGAGGTTGAT |
| *pyrosequencing primers* | |  |
| FTL1 | FTL1 F1 | GGTGGTAGTTTGTTATTTAAGATGAGTGA |
|  | FTL1 R1 | ACAATCCCTTATATTACATCCCTCTA |
|  | FTL1 S1 | ATTATGTTTTTATTATTTTAAAGGT |
|  | FTL1 F2 | AGGTTGGGTAATTTAAAGTTGTTATAT |
|  | FTL1 R2 | AAACAATAACTCCCACAATCTTCCTTACC |
|  | FTL1 S2 | GAGAGGTTTAGGTTATTTTATATAG |
| IGF2BP1 | IGF2BP1 F1 | TGGGGGAGGATGGTTTAGTT |
|  | IGF2BP1 R1 | ACCCCAAAATCTTCCACTTCTCTTTATCTA |
|  | IGF2BP1 S1 | CTTCCACTTCTCTTTATCTATCTC |
|  | IGF2BP1 F2 | TGGGTGGGGGTTGTTATT |
|  | IGF2BP1 R2 | CCCACCCCAAAAATTATCCC |
|  | IGF2BP1 S2 | GGTTTAGGATAGAAGGGAAGAAG |

Supplementary Table 3. The methylation levels of CpG sites in the promoter regions of FLT1 and IGF2BP1 were detected by pyrosequencing.

| Gene | Site | Medical abortion samples (controls, n=46) | Spontaneous abortion samples (cases, n=31) | P Value |
| --- | --- | --- | --- | --- |
| FLT1 | Site1 | 23.04±0.92 | 22.28±1.34 | 0.959 |
|  | Site2 | 36.15±1.25 | 33.99±2.39 | 0.622 |
|  | Site3 | 21.33±0.65 | 19.8±0.97 | 0.220 |
|  | Site4 | 44.81±1.47 | 42.13±1.88 | 0.170 |
|  |  |  |  |  |
| IGF2BP1 | Site1 | 17.72±0.73 | 19.38±1.27 | 0.568 |
|  | Site2 | 13.17±0.38 | 11.18±0.53 | 0.003 |
|  | Site3 | 18.49±0.76 | 17.14±1.03 | 0.266 |
|  | Site4 | 20.96±0.65 | 17.32±1.19 | 0.006 |
|  | Site5 | 13.52±0.37 | 13.51±0.87 | 0.200 |
|  | Site6 | 8.8±0.6 | 8.71±0.67 | 0.655 |
|  | Site7 | 10.49±0.39 | 9.07±0.47 | 0.019 |
|  | Site8 | 13.59±0.71 | 10.93±0.43 | 0.002 |
|  | Site9 | 9.8±0.32 | 8.6±0.54 | 0.037 |
|  | Site10 | 4.81±0.57 | 2.4±0.72 | 0.010 |
|  | Site11 | 9.42±0.93 | 7.91±1.63 | 0.080 |
|  | Site12 | 3.11±0.59 | 1.63±0.86 | 0.033 |

Supplementary Table 4. Descriptive indices of air pollutants and climate factors in the city Tianjin from November 1, 2013 to December 31, 2018 (reported as per day).

| Variable | n | Mean | Median | Minimum | Maximum | SEM | SD |
| --- | --- | --- | --- | --- | --- | --- | --- |
| PM2.5 (μg/m^3^) | 1885 | 68.35 | 54.00 | 7.00 | 383.00 | 1.19 | 2672.73 |
| PM10 (μg/m^3^) | 1885 | 109.16 | 91.00 | 11.00 | 483.00 | 1.59 | 4758.80 |
| CO (mg/m^3^) | 1885 | 1.38 | 1.20 | 0.30 | 9.00 | 0.02 | 0.62 |
| NO2 (μg/m^3^) | 1885 | 47.80 | 43.00 | 4.00 | 176.00 | 0.53 | 532.04 |
| SO2 (μg/m^3^) | 1885 | 27.77 | 17.00 | 2.00 | 260.00 | 0.72 | 970.76 |
| O3 (μg/m^3^) | 1885 | 87.83 | 76.00 | 3.00 | 265.00 | 1.27 | 3030.73 |

Supplementary Table 5: PLS-PM analysis for the relationships between latent variables.

|  | Path Coefficients | Estimated p-value | SE* | 95% CI |
| --- | --- | --- | --- | --- |
| 𝛽 _Air pollution -> IGF2BP1 methylation_ | -0.259 | 0.004 | 0.091 | (-0.460, -0.098) |
| 𝛽 _Air pollution -> RSA_ | 0.287 | 0.004 | 0.100 | (0.059, 0.453) |
| 𝛽 _IGF2BP1 methylation ->RSA_ | -0.361 | <0.001 | 0.096 | (-0.567, -0.199) |

* SE: Standard Error.
