# Supplementary material for: Integrated single-cell RNA-seq and DNA methylation reveal the effects of air pollution in patients with recurrent spontaneous abortion
Source: Clin Epigenetics. 2022 Aug 23;14:105. doi: 10.1186/s13148-022-01327-2 (PMC9400245; doi:10.1186/s13148-022-01327-2)
Supplement: Supplementary file 3 — Additional file 3: Fig. 1. Cell communication analysis between 14 cluster cells. Circle plot shows the number of interactions and interaction weights/strength of 14 cluster cells between case and control. [file 13148_2022_1327_MOESM3_ESM.pdf]

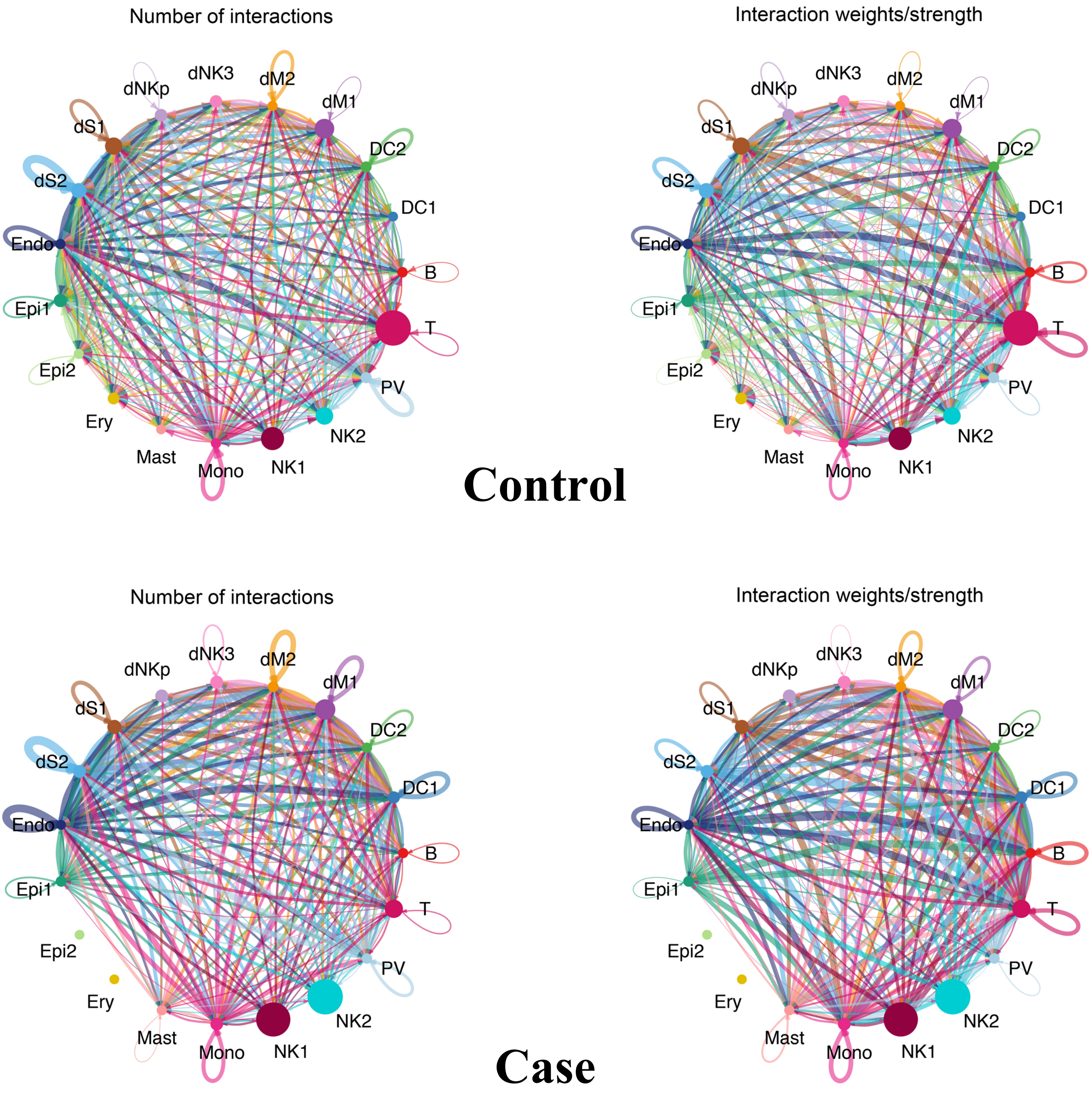

Number of interactions
Interaction weights/strength
dNK3
dM2
dNKp
dM1
dS1
DC2
dS2
DC1
Endo
B
Epi1
T
Epi2
PV
Ery
NK2
Mast
Mono
NK1
Control
Number of interactions
Interaction weights/strength
dNK3
dM2
dNKp
dM1
dS1
DC2
dS2
DC1
Endo
B
Epi1
T
Epi2
PV
Ery
NK2
Mast
Mono
NK1
Case
