# Supplementary figures and images for: Integrated single-cell RNA-seq and DNA methylation reveal the effects of air pollution in patients with recurrent spontaneous abortion
Source: Clin Epigenetics. 2022 Aug 23;14:105. doi: 10.1186/s13148-022-01327-2 (PMC9400245; doi:10.1186/s13148-022-01327-2)

**A**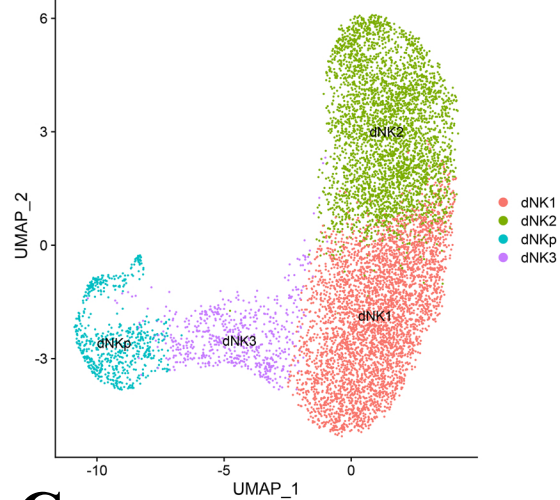**B**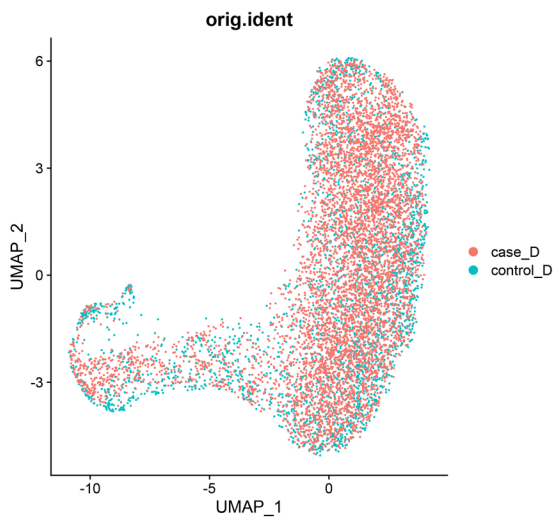**C**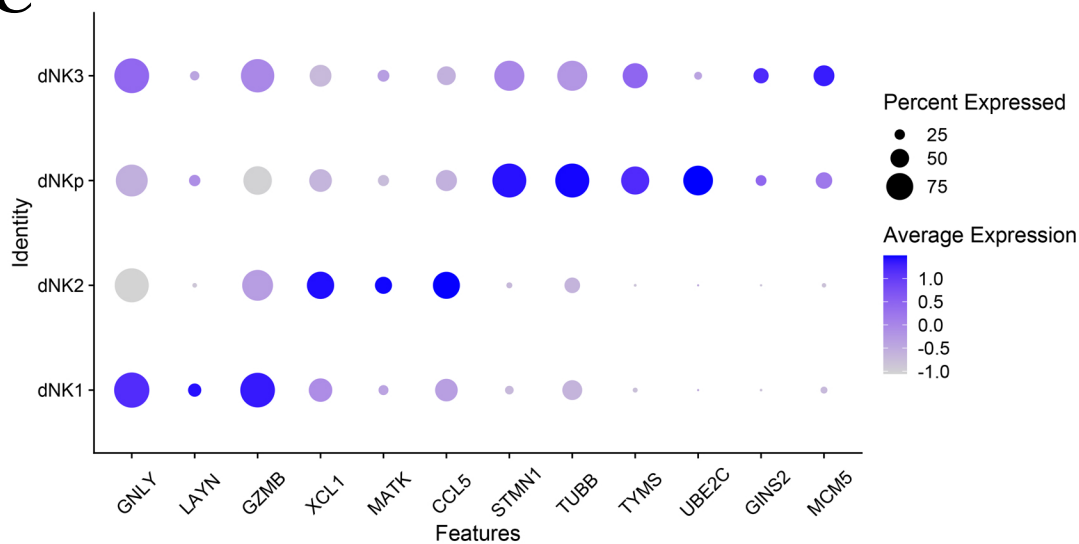**D**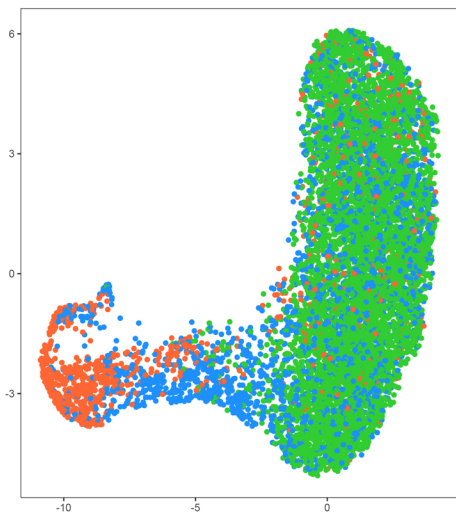**E**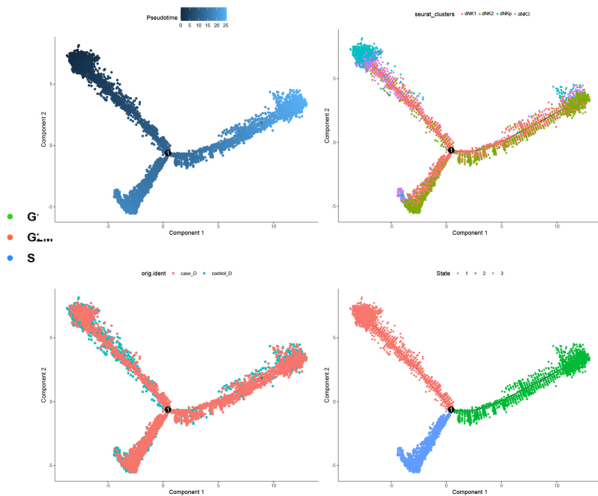

Supplement: Supplementary file 4 — Additional file 4: Fig. 2. Molecular details and subclusters of dNKs were revealed by scRNA-seq. (A) A UMAP projection of the dNKs from one RSA patient and one matched healthy control. Different colors indicate cell clusters. (B) Dot plot shows the expression of marker genes for each subcluster of dNK. (C) Dot plot shows the expression of cell cycle-related genes. (D) Developmental trajectories of dNK subsets, cells colored by conditions of trajectories state, groups, subclusters, and pseudotime. [file 13148_2022_1327_MOESM4_ESM.pdf]

**A**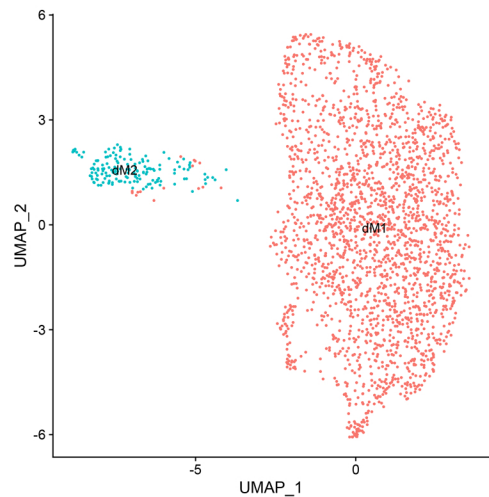**B**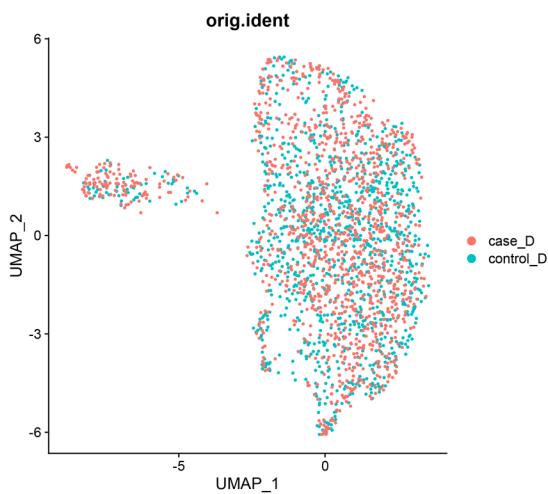**C**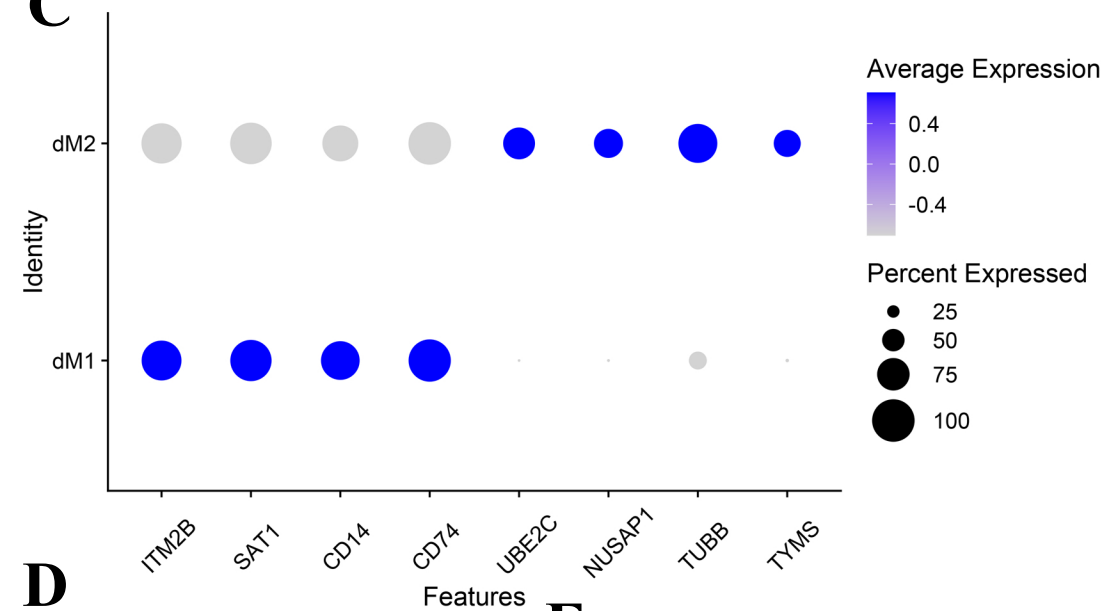**D**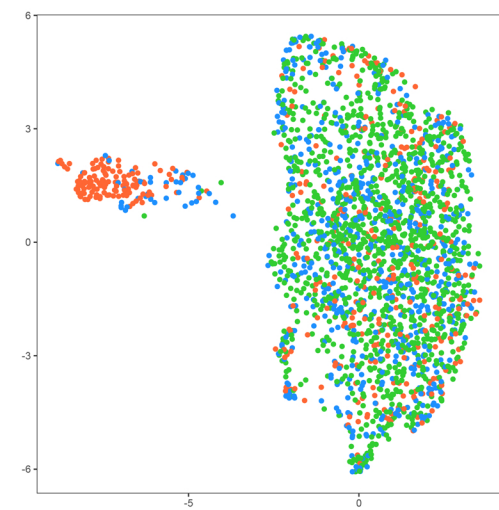**E**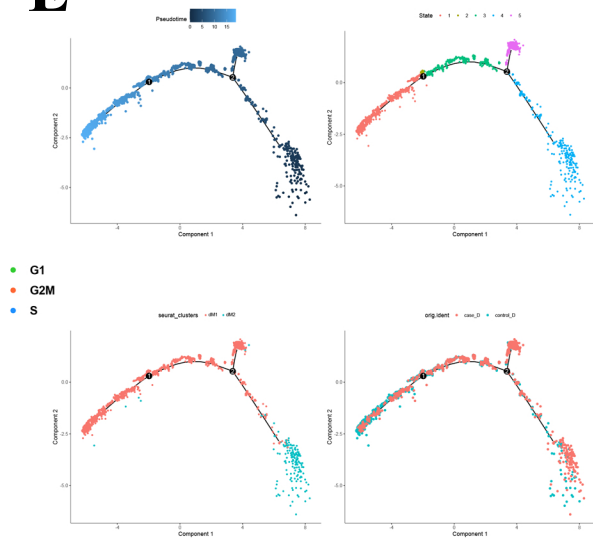

Supplement: Supplementary file 5 — Additional file 5: Fig. 3. Molecular details and subclusters of dM were revealed by scRNA-seq. (A) A UMAP projection of the dM from three RSA patients and three matched healthy controls. Different colors indicate cell clusters. (B) Dot plot shows the expression of marker genes for each subcluster of dM. (C) Dot plot shows the expression of cell cycle-related genes. (D) Developmental trajectories of dM subsets, cells colored by conditions of trajectories state, groups, subclusters, and pseudotime. [file 13148_2022_1327_MOESM5_ESM.pdf]

A

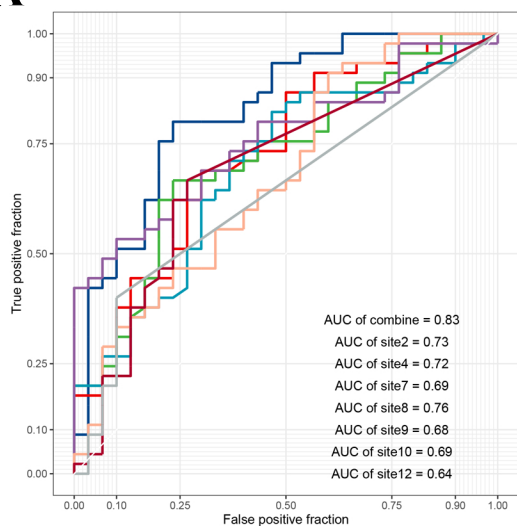

B

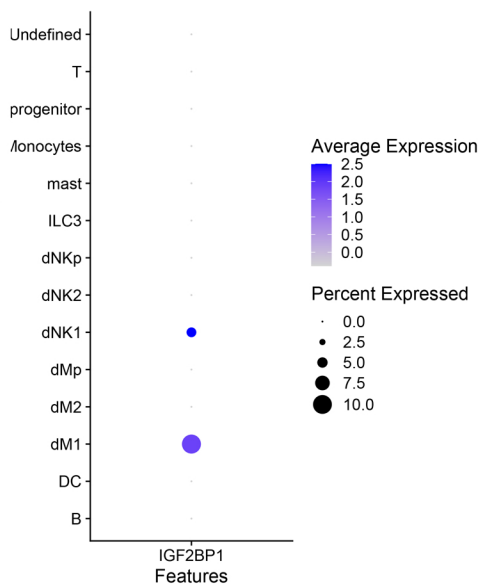

C

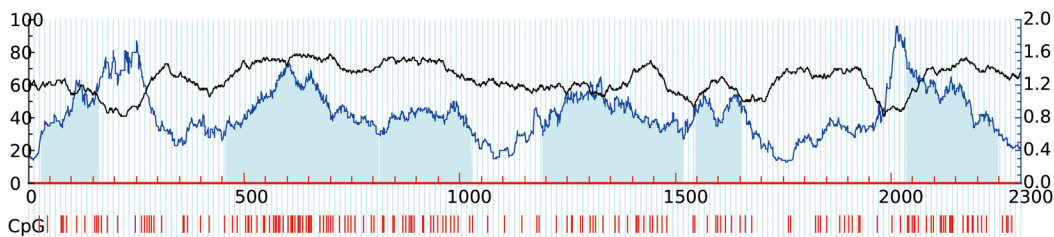

## IGF2BP1 Promoter

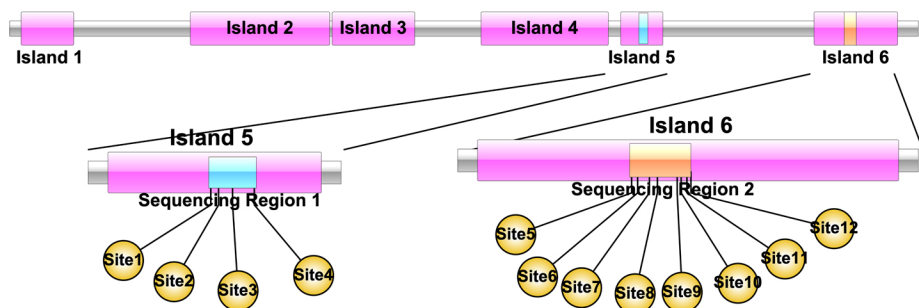

D

MAZ

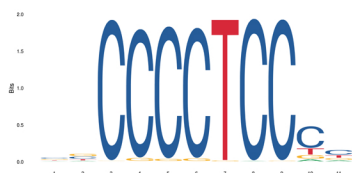

VEZF1

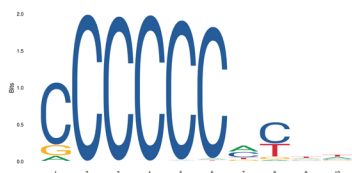

ZNF148

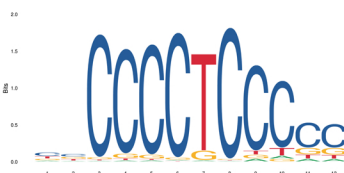

SP1

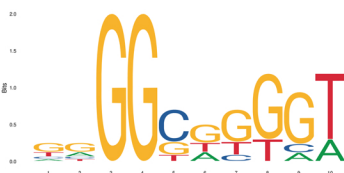

KLF5

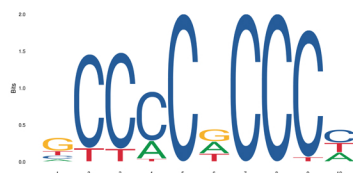

TFAP2C(var.2)

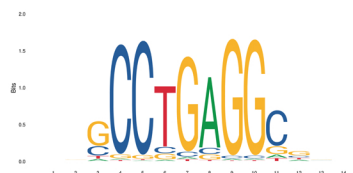

Supplement: Supplementary file 6 — Additional file 6: Fig. 4. Analysis of IGF2BP1 expression and its promoter region. (A) ROC analysis of methylation of IGF2BP1 in patient with RSA and controls. (B) scRNA-seq analysis of the expression of IGF2BP1 in 14 cell subsets. (C) Distribution of CpG island in the IGF2BP1 promoter region. (D) Prediction of transcription factors binding to each CpG island. [file 13148_2022_1327_MOESM6_ESM.pdf]

A

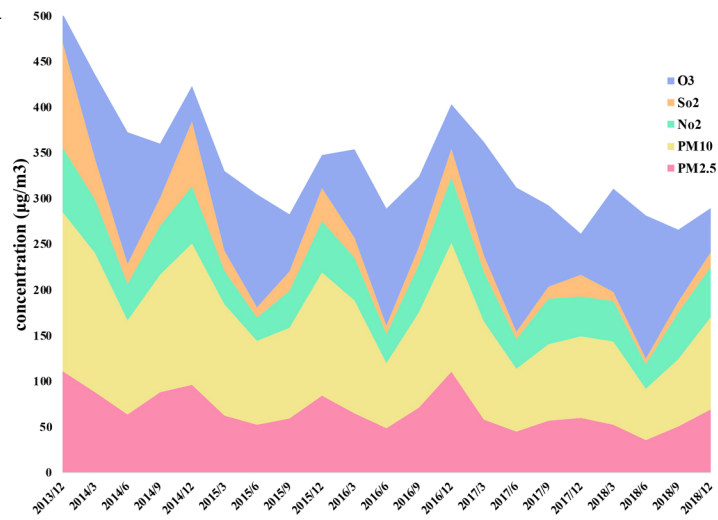

B

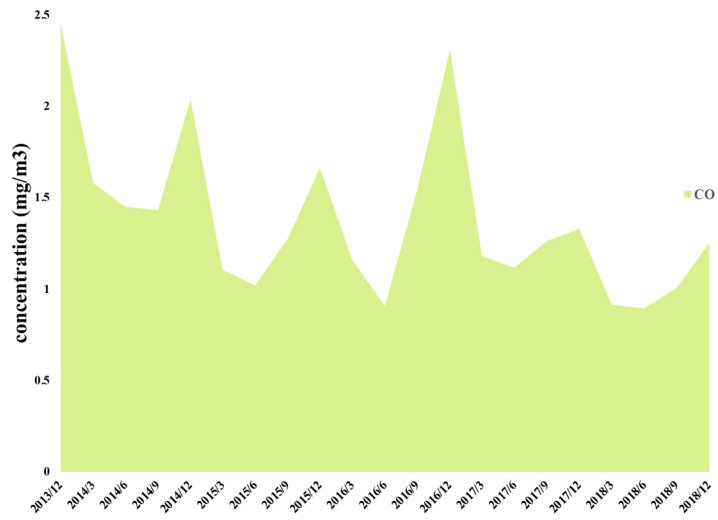

C

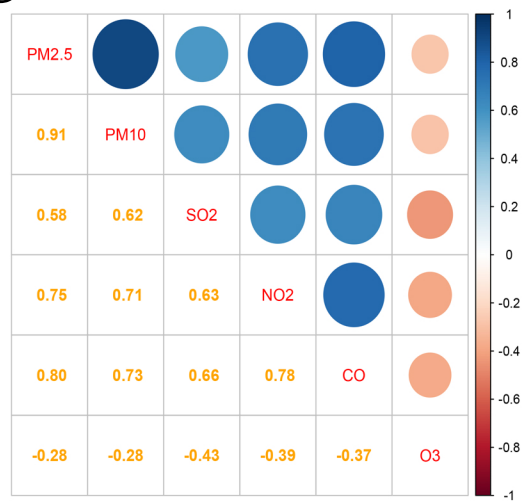

Supplement: Supplementary file 7 — Additional file 7: Fig. 5. The relationship between air pollution-related genes and RSA. (A) and (B) Monthly air pollutant concentration curve from 2014 to 2018. (C) Correlation heatmap plot shows the association between six air pollutants. [file 13148_2022_1327_MOESM7_ESM.pdf]
